# Erythropoietin, ferritin, haptoglobin, hemoglobin and transferrin receptor in metabolic syndrome: a case control study

**DOI:** 10.1186/1475-2840-11-116

**Published:** 2012-09-27

**Authors:** Päivi Hämäläinen, Juha Saltevo, Hannu Kautiainen, Pekka Mäntyselkä, Mauno Vanhala

**Affiliations:** 1 Department of Internal Medicine, Tampere University Hospital, Teiskontie, 35 33521, Tampere, Finland; 2 Department of Medicine, Central Finland Central Hospital, Jyväskylä, Finland; 3 Unit of Family Practice, Central Finland Central Hospital, Jyväskylä, and Unit of Primary Health Care, Kuopio University Hospital, Kuopio, Finland; 4 Unit of Primary Health Care, University of Eastern Finland, and Kuopio University Hospital, Kuopio, Finland; 5 Unit of Family Practice of Central Finland Central Hospital, Jyväskylä and University of Eastern Finland and Kuopio University Hospital, Kuopio, Finland

**Keywords:** Erythropoietin, Ferritin, Hemoglobin, Metabolic syndrome

## Abstract

**Background:**

Increased ferritin concentrations are associated with metabolic syndrome (MetS). The association between ferritin as well as hemoglobin level and individual MetS components is unclear. Erythropoietin levels in subjects with MetS have not been determined previously. The aim of this study was to compare serum erythropoietin, ferritin, haptoglobin, hemoglobin, and transferrin receptor (sTFR) levels between subjects with and without MetS and subjects with individual MetS components.

**Methods:**

A population based cross-sectional study of 766 Caucasian, middle-aged subjects (341 men and 425 women) from five age groups born in Pieksämäki, Finland who were invited to a health check-up in 2004 with no exclusion criteria. Laboratory analyzes of blood samples collected in 2004 were done during year 2010. MetS was defined by National Cholesterol Education Program criteria.

**Results:**

159 (53%) men and 170 (40%) women of study population met MetS criteria. Hemoglobin and ferritin levels as well as erythropoietin and haptoglobin levels were higher in subjects with MetS (p < 0.001, p = 0.018). sTFR level did not differ significantly between subjects with or without MetS. Hemoglobin level was significantly higher in subjects with any of the MetS components (p < 0.001, p = 0.002). Ferritin level was significantly higher in subjects with abdominal obesity or high TG or elevated glucose or low high density cholesterol component (p < 0.001, p = 0.002, p = 0.02). Erythropoietin level was significantly higher in subjects with abdominal obesity component (p = 0.015) but did not differ significantly between subjects with or without other MetS components. Haptoglobin level was significantly higher in subjects with blood pressure or elevated glucose component o MetS (p = 0.028, p = 0.025).

**Conclusion:**

Subjects with MetS have elevated hemoglobin, ferritin, erythropoietin and haptoglobin concentrations. Higher hemoglobin levels are related to all components of MetS. Higher ferritin levels associate with TG, abdominal obesity, elevated glucose or low high density cholesterol. Haptoglobin levels associate with blood pressure or elevated glucose. However, erythropoietin levels are related only with abdominal obesity. Higher serum erythropoietin concentrations may suggest underlying adipose tissue hypoxemia in MetS.

## 

Metabolic syndrome (MetS) is a pathophysiological disorder with clustering of risk factors -abdominal obesity, increased blood pressure, glucose intolerance and dyslipidemia - for cardiovascular disease and type 2 diabetes [1,2]. Previous studies have reported alterations in hematological parameters and iron metabolism: a trend towards higher hemoglobin concentrations and serum ferritin levels in subjects with MetS [3-6]. However, the association between hemoglobin as well as ferritin level and individual MetS components is still unclear.

Erythropoietin (EPO) is a glycoprotein hormone whose production in kidneys is stimulated by hypoxia and it is a known stimulator of erythrocyte production and hemoglobin synthesis [7]. Recently, increasing amount of evidence has suggested that reduced adipose tissue oxygenation and cellular hypoxia may be an underlying cause of adipose tissue dysfunction contributing to metabolic changes like insulin resistance associated with obesity and MetS [8-10]. Erythropoietin levels in subjects with MetS have not been determined previously.

The aim of this study was to compare serum haptoglobin, hemoglobin, ferritin, erythropoietin and transferrin receptor levels between subjects with and without MetS and extend these findings to include individual MetS components.

## Research design and methods

### Study sample

The study population primarily consisted of 1294 middle-aged subjects from Pieksämäki, Finland, who were born in 1942, 1947, 1952, 1957 or 1962 and invited to a health check-up in the years 1997–1998 initially and to a follow-up check-up in 2004. A total of 923 out of 1294 subjects participated in the initial examination in 1997–1998 and 766 of these participated in a second health check-up in 2003–2004 when hematological laboratory tests were taken. These hematological laboratory tests were analyzed in Kuopio University laboratory during year 2010.

The final analysis included data from these 766 subjects. The study protocol was approved by the Ethics Committee of Kuopio University Hospital and the University of Eastern Finland. All participants gave informed written consent.

All subjects filled in a questionnaire about their smoking habits, alcohol consumption, and physical activity. They were also interviewed by a trained nurse. Subjects who smoked daily were considered to be current smokers. Alcohol consumption was divided into three categories: low, meaning no alcohol use; moderate (less than two portions of alcohol per day); and high (more than two portions per day). Physical activity was considered to be high in subjects who exercised daily at least 30 minutes in their leisure time, moderate in subjects who exercised at least three times per week, and low if exercising frequency was less than three times per week [11].

Two trained nurses performed the study processing and physical examination. Blood pressure was measured with a mercury sphygmomanometer in a sitting position after 15 minutes of rest. The measurement was repeated after five minutes. The mean of the two measurements was used in the statistical analyses. Waist circumference was measured from the midpoint between the lateral iliac crest and the lowest rib to an accuracy of 0.5 cm.

MetS was defined by the updated National Cholesterol Education Program Expert Panel on Detection, Evaluation and Treatment of High Blood Cholesterol in Adults (ATP III) criteria [12]. Subjects with three or more of the following components were classified as having MetS: (1) increased waist circumference (≥102 cm (≥40 in) for men and ≥88 cm (≥35 in) for women); (2) elevated fasting total triglycerides (≥1.7 mmol/l (≥150 mg/dl) or treatment for dyslipidemia); (3) low fasting serum high density lipoprotein (HDL) cholesterol (<1.03 mmol/l (<40 mg/dl) in men or <1.29 mmol/l (<50 mg/dl) in women or treatment for dyslipidemia); (4) systolic blood pressure ≥130 mmHg or diastolic blood presure ≥85 mmHg or the use of antihypertensive medication; and (5) fasting plasma glucose ≥5.6 mmol/l (≥100 mg/dl) or the use of antihyperglycemic medication.

### Laboratory methods

Fresh serum samples were drawn after an overnight fast. Plasma was separated by centrifugation for the determination of glucose and lipids and the samples were frozen immediately and stored at - 70°C.

Samples were analyzed in Kuopio regional laboratory during year 2010.

Plasma glucose concentration was measured by an automated colorimetric method (Peridochrom Glucose GOD-PAP, Boehringer, Germany). Serum triglycerides were measured from fresh serum samples by enzymatic colorimetric methods (CHOD-PAP, GPO-PAP, Boehringer Mannheim GmbH, Germany). Serum HDL cholesterol was measured by the same method after precipitation of low-density lipoprotein cholesterol and very low-density lipoprotein cholesterol with phosphotungestic acid and magnesium. High-sensitivity C-reactive protein (hs-CRP) was measured with an Immulite analyzer and a DPC high-sensitivity CRP assay (DPL, Los Angeles, CA, USA).

White blood cell and platelet count, hemoglobin and hematocrit were measured using an automatic electronic cell calculator. Serum EPO was analyzed using an immunoluminometric assay method. Serum soluble transferrin receptor concentration was measured by a particle enhanced immunoturbidimetric assay (Cobas c systems, Roche Diagnostics GmbH, Mannheim, Germany). Serum ferritin concentration was analyzed using an electrochemiluminescence immunoassay (Roche Diagnostics GmbH, Mannheim, Germany).

The analytical method for plasma haptoglobin concentration measurements was an immunoturbidimetric assay (Cobas c systems, Roche Diagnostics GmbH, Mannheim, Germany, ACN 228). Serum creatinine was measured using an enzymatic method.

### Statistical analyses

The results are expressed as means and standard deviations (SDs) for continuous variables and as proportions for categorical variables. The normality of variables was evaluated by the Shapiro-Wilk test. Statistical comparisons between the groups were performed using the chi-square test, *t*-test, or bootstrap-type *t*-test as appropriate. Bootstrap type analysis of covariance was also used to compare the groups as measurements. In these analyses age values, sex, smoking, physical activity and hs-CRP values were used as covariates. For all analyses, P < 0.05 was considered significant.

## Results

The study population included 425 women and 341 men with a mean age of 52.1 ± 6.4 and 52.1 ± 6.2 years, respectively. MetS was present in 52% of women and in 48% of men. Table 1 shows the clinical and lifetime factors for subjects with and without MetS. Both women and men with MetS were significantly older than subjects without. Life-style factors did not differ significantly between subjects with or without MetS. In those with MetS, 14% of female and 26% of male subjects were classified as current smokers. In those without MetS, the proportions of female and male smokers to non-smokers were 19% and 25% respectively.

**Table 1:** **Clinical and life-style characteristicsof the study populationin subjects with andwithout the MetS**

**Characteristics**	**Male**		**p**	**Female**		**p**
	**MetS present**	**MetS not present**		**MetS present**	**MetS not present**	
	**N = 159**	**N = 182**		**N = 170**	**N = 255**	
Age, years **	53.7 (5.8)	50.7 (6.2)	<0.001	54.4 (5.7)	50.5 (6.4)	<0.001
Body mass index **	29.8 (3.9)	25.2 (2.5)	<0.001	30.8 (5.3)	25.1 (3.5)	<0.001
Waist, cm **	103.8 (10.7)	89.6 (7.2)	<0.001	96.2 (12.4)	81.0 (8.5)	<0.001
FP-gluc mmol/L **	6.5 (1.4)	5.8 (0.8)	<0.001	6.3 (1.5)	5.5 (0.4)	<0.001
BP systolic, mmHg **	143 (19)	136 (17)	<0.001	144 (17)	131 (16)	<0.001
BP diastolic, mmHg **	87 (9)	82 (10)	<0.001	86 (9)	79 (8)	<0.001
HDL-C, mmol/L **	1.3 (0.4)	1.6 (0.4)	<0.001	1.6 (0.4)	1.8 (0.3)	<0.001
Trigly, mmol/L **	1.9 (1.5)	1.1 (0.5)	<0.001	1.6 (0.8)	1.0 (0.3)	<0.001
Creatinine μmol/L **	88.5 (11.9)	85.9 (8.7)	0.02	75.3 (14.8)	73.8 (8.9)	0.17
Hemoglobin (g/L) **	154 (9)	150 (9)	<0.001*	141 (10)	136 (9)	<0.001*
Ferritin (μg/L) **	216 (165)	151 (112)	<0.001*	94 (75)	61 (48)	<0.001*
TFR (mg/L) **	2.9 (2.8)	2.6 (0.6)	0.12*	2.8 (0.9)	2.7 (1.0)	0.32*
Haptoglobin (g/L) **	1.3 (0.6)	1.1 (0.5)	0.012*	1.4 (0.6)	1.2 (0.4)	<0.001*
Hs-CRP (mg/L) **	2.4 (3.5)	1.5 (2.9)	0.058*	3.1 (3.4)	1.5 (2.3)	<0.001*
Components of MetS:						
Waist n (%)	102 (64)	8 (4)	<0.001	134 (79)	40 (16)	<0.001
FP-glucose n (%)	144 (91)	113 (62)	<0.001	139 (83)	85 (33)	<0.001
Blood pressure n (%)	146 (92)	119 (65)	<0.001	154 (90)	126 (49)	<0.001
HDL-cholesterol n (%)	90 (57)	2 (1)	<0.001	101 (59)	10 (4)	<0.001
Triglycerides n (%)	116 (73)	16 (9)	<0.001	118 (69)	6 (2)	<0.001
Life-style factors, n (%):						
Current smoker	42 (26)	46 (25)	0.81	25 (14)	49 (19)	0.23
Current use of alcohol						0.038
Low (nothing)	20 (12)	26 (14)	0.055	46 (28)	46 (18)	
Moderate	68 (43)	97 (54)	0.055	97 (58)	177 (70)	
High	71 (45)	58 (32)	0.055	24 (14)	31 (12)	
Physical activity n (%)						0.83
Low	33 (21)	56 (31)	0.11	55 (33)	80 (32)	
Moderate	97 (61)	96 (53)	0.11	92 (55)	146 (57)	
High	29 (18)	30 (16)	0.11	21 (12)	28 (11)	

*Values are adjusted for age.

** Mean (SD).

Abbreviations: Fp-gluc, fasting plasma glucose; BP systolic, systolic blood pressure; BP diastolic, diastolic blood pressure; HDL-C, high density cholesterol; Trigly, triglycerides; TFR, transferrin receptor; Hs-CRP, high sensitivity c-reactive protein.

Components of MetS.

1. Waist >102 cm (male) or >88 cm (female).

2. FP-glucose ≥ 5.6 mmol/L.

3. Systolic blood pressure ≥ 130 mmHg or diastolic ≥ 85 mmHg or antihypertensive medication.

4. HDL–cholesterol < 1.03 mmol/l (men) or < 1.29 mmol/l (women) or medication for dyslipidemia.

5. Triglycerides >1.7 mmol/l or medication for dyslipidemia.

Current use of alcohol was consireded low with no use of alcohol, moderate with use of <2 portions/day and high with use of >2 portions/day. Physical activity was considered to be high in subjects who exercised daily at least 30 minutes in their leisure time, moderate in subjects who exercised at least three times per week, and low if exercising frequency was less than three times per week.

Blood pressure was the most common component of MetS. It was present in 78% of the men and 66% of the women. High fasting plasma glucose was also present in a large part of the subject (75% of the men and 53% of the men, Table 1).

**Figure 1:**
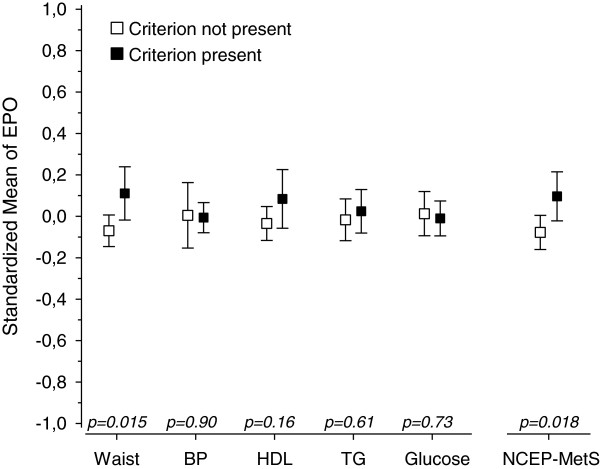
**Eryhtropoietin mean values and individual MetS components. **All values are standardized for age, sex, hs-CRP, smoking and physical activity. Abbreviations: BP, blood pressure; EPO, erythropoietin; HDL, high density lipoprotein cholesterol; TFR, transferrin receptor; TG, triglycerides. NCEP (National Cholesterol Education Program Expert Panel on Detection, Evaluation and Treatment of High Blood Cholesterol in Adults) criteria of MetS: Waist >102 cm(male) or >88 cm(female); FP-glucose ≥ 5.6 mmol/L; Systolic blood pressure ≥ 130 mmHg or diastolic ≥ 85 mmHg or antihypertensive medication; HDL–cholesterol < 1.03 mmol/l (men) or < 1.29 mmol/l (women) or medication for dyslipidemia; triglycerides >1.7 mmol/l or medication for dyslipidemia.

**Figure 2:**
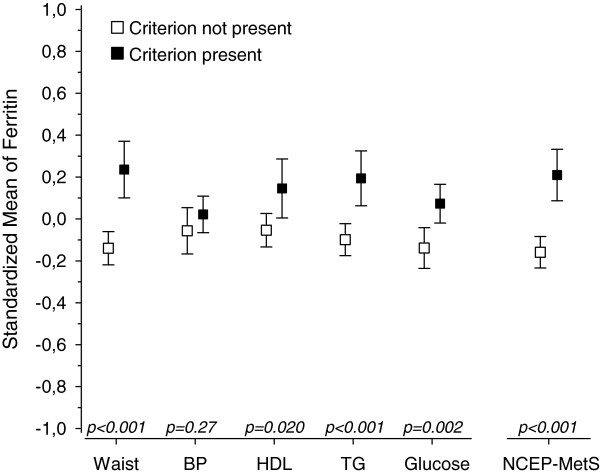
**Ferritin mean values and individual MetS components. **All values are standardized for age, sex, hs-CRP, smoking and physical activity. Abbreviations: BP, blood pressure; EPO, erythropoietin; HDL, high density lipoprotein cholesterol; TFR, transferrin receptor; TG, triglycerides. NCEP (National Cholesterol Education Program Expert Panel on Detection, Evaluation and Treatment of High Blood Cholesterol in Adults) criteria of MetS: Waist >102 cm(male) or >88 cm(female); FP-glucose ≥ 5.6 mmol/L; Systolic blood pressure ≥ 130 mmHg or diastolic ≥ 85 mmHg or antihypertensive medication; HDL–cholesterol < 1.03 mmol/l (men) or < 1.29 mmol/l (women) or medication for dyslipidemia; triglycerides >1.7 mmol/l or medication for dyslipidemia.

**Figure 3:**
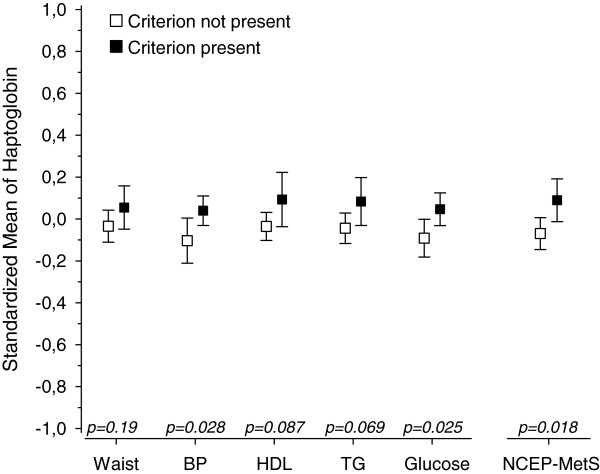
**Haptoglobin mean values and individual MetS components. **All values are standardized for age, sex, hs-CRP, smoking and physical activity. Abbreviations: BP, blood pressure; EPO, erythropoietin; HDL, high density lipoprotein cholesterol; TFR, transferrin receptor; TG, triglycerides. NCEP (National Cholesterol Education Program Expert Panel on Detection, Evaluation and Treatment of High Blood Cholesterol in Adults) criteria of MetS: Waist >102 cm(male) or >88 cm(female); FP-glucose ≥ 5.6 mmol/L; Systolic blood pressure ≥ 130 mmHg or diastolic ≥ 85 mmHg or antihypertensive medication; HDL–cholesterol < 1.03 mmol/l (men) or < 1.29 mmol/l (women) or medication for dyslipidemia; triglycerides >1.7 mmol/l or medication for dyslipidemia.

**Figure 4:**
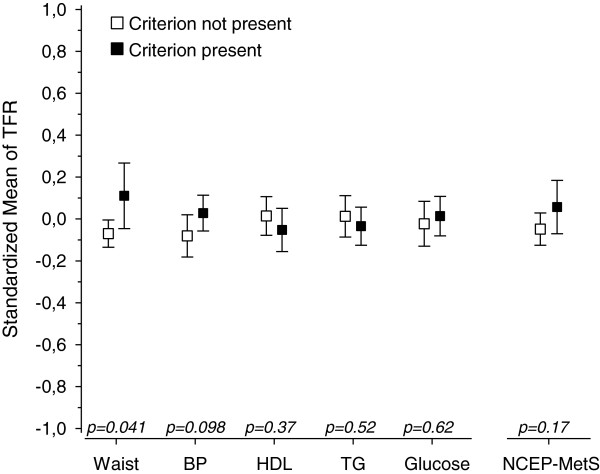
**Transferrin receptor mean values and individual MetS components. **All values are standardized for age, sex, hs-CRP, smoking and physical activity. Abbreviations: BP, blood pressure; EPO, erythropoietin; HDL, high density lipoprotein cholesterol; TFR, transferrin receptor; TG, triglycerides. NCEP (National Cholesterol Education Program Expert Panel on Detection, Evaluation and Treatment of High Blood Cholesterol in Adults) criteria of MetS: Waist >102 cm(male) or >88 cm(female); FP-glucose ≥ 5.6 mmol/L; Systolic blood pressure ≥ 130 mmHg or diastolic ≥ 85 mmHg or antihypertensive medication; HDL–cholesterol < 1.03 mmol/l (men) or < 1.29 mmol/l (women) or medication for dyslipidemia; triglycerides >1.7 mmol/l or medication for dyslipidemia.

**Figure 5:**
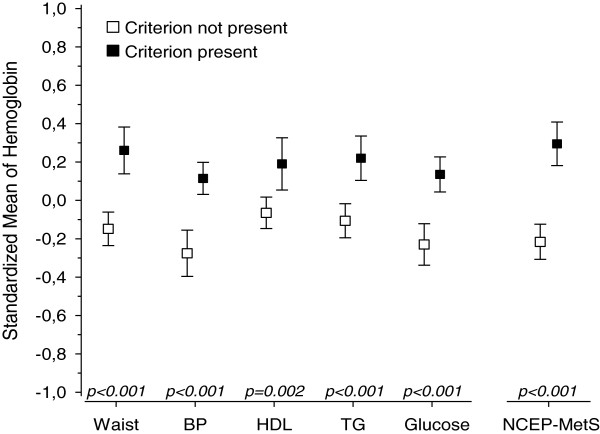
**Hemoglobin mean values and individual MetS components. **All values are standardized for age, sex, hs-CRP, smoking and physical activity. Abbreviations: BP, blood pressure; EPO, erythropoietin; HDL, high density lipoprotein cholesterol; TFR, transferrin receptor; TG, triglycerides. NCEP (National Cholesterol Education Program Expert Panel on Detection, Evaluation and Treatment of High Blood Cholesterol in Adults) criteria of MetS: Waist >102 cm(male) or >88 cm(female); FP-glucose ≥ 5.6 mmol/L; Systolic blood pressure ≥ 130 mmHg or diastolic ≥ 85 mmHg or antihypertensive medication; HDL–cholesterol < 1.03 mmol/l (men) or < 1.29 mmol/l (women) or medication for dyslipidemia; triglycerides >1.7 mmol/l or medication for dyslipidemia.

Figures 1, 2, 3, 4 and 5 show standardized means for erythropoietin, ferritin, haptoglobin, serum transferrin receptor (sTFR) and hemoglobin in subjects with and without MetS and in subjects with or without an individual MetS component. All results are standardized for age, sex, smoking, physical activity and hs-CRP. Also adjustment for creatinine as a marker of renal function and for alcohol comsumption was done afterwards (data not shown). This did not change the results. Mean hemoglobin and mean ferritin were significantly higher in subjects with MetS (p < 0.001). Mean erythropoietin was significantly higher in men with MetS (p = 0.006) and remained significantly higher in subjects with MetS after adjusting for sex (p = 0.018). Mean haptoglobin was significantly higher in subjects with MetS (p = 0.018). Mean sTFR did not differ significantly between subjects with or without MetS.

Mean hemoglobin was significantly higher in subjects with any of the MetS components (abdominal obesity, blood pressure (BP), low HDL, high triglycerides (TG) or elevated glucose). Mean ferritin was significantly higher in subjects with abdominal obesity or low HDL or high TG or elevated glucose component. Mean erythropoietin was significantly higher in subjects with abdominal obesity component but did not differ significantly between subjects with or without other components of MetS. Mean haptoglobin was significantly higher in subjects with blood pressure or elevated glucose component. Mean sTFR was significantly higher in subjects with abdominal obesity component but did not differ significantly between subjects with or without other components of MetS.

## Discussion and conclusion

To our knowledge, this is the first study to show higher hemoglobin, serum ferritin, haptoglobin and also erythropoietin levels in subjects with MetS and extending these findings to include individual MetS components.

Recently, increasing evidence has suggested that reduced adipose tissue oxygenation and cellular hypoxia may be an underlying cause of adipose tissue dysfunction contributing to metabolic changes associated with obesity and MetS [8-10]. It was demonstrated that hypoxia creates an insulin resistant state in human adipocytes by inhibiting phosphorylation of the insulin receptor, leading to a decrease in glucose transport [10]. Insulin resistance has been the most accepted and unifying hypothesis to describe the pathophysiology of the metabolic syndrome [1].

Also, previous studies have shown reduced adipose tissue oxygenation in obese compared to normal-weight subjects and EPO gene transcription stimulating factor (HIF-1) over-expression in the adipose tissue of obese subjects [8][13]. Hypoxia is a known stimulator of erythropoietin production as well as EPO is a stimulator of hemoglobin synthesis [7]. EPO levels were significantly higher in subjects with MetS as well as in subjects with MetS abdominal obesity component in this study which may suggest underlying adipose tissue hypoxia in MetS.

Hemoglobin levels were significantly higher in subjects with MetS or with any of the components of MetS. This is supported by a previous study of working-age thai subjects that showed increased hemoglobin concentrations with increasing numbers of MetS components but only in women [14].

Further research is needed to investigate possible association between higher hemoglobin and EPO levels in subjects with MetS.

Previously, it was shown that nocturnal intermittent hypoxia, a marker for obstructive sleep apnea (OSA), is positively associated with MetS and its components [15]. One previous study reported lower serum EPO concentrations after continuous positive pressure airway treatment in patients with OSA [16]. Also, higher serum EPO concentrations have been reported in patients with central sleep apnea and nocturnal hypoxia compared to healthy controls [17].

Previous studies have shown associations between serum ferritin or sTFR and increased risk of type 2 diabetes [18-21]. Recently, it was also shown that single nucleotide polymorphism (SNP) in genes that are related to body iron status are associated with risk of type 2 diabetes (T2D). SNP in gene that was related high sTFR levels and low ferritin levels was associated with lower risk of T2D, as well [20].

The finding that subjects with MetS had significantly higher serum ferritin levels supports previous results [3-6]. In addition, ferritin levels were significantly higher in subjects with abdominal obesity or high TG or elevated glucose or low high-density cholesterol MetS component but not in subjects with blood pressure component. Previous studies have shown that higher serum ferritin concentrations are associated with increased TG concentration in men and with elevated glucose in women [3,4].

Because ferritin is an acute phase reactant, all results were adjusted for hs-CRP to estimate the impact of inflammation. Ferritin levels remained significantly higher after hs-CRP standardization suggesting that mechanisms other than inflammation may be influencing ferritin concentration in the subjects with MetS. However, we were not able to estimate other markers of inflammation or level of proinflammatory cytokines like tumor necrosis factor alfa and interleukins in this study.

Higher hs-CRP levels have also previously shown to be associated with MetS and its separate components as well as median hs-CRP levels to be increased with increasing number of MetS components [21-24]. In addition, the degree of central obesity seemed to be the main determinant of an increased hs-CRP level [24]. In our study hs-CRP levels were significantly higher in women with MetS and almost significantly higher in men with MetS compared those without (Table 1).

Under hypoxia and also when erythropoiesis is stimulated, human iron-regulatory hormone, hepcidin, production is suppressed [25,26]. Theoretically, suppression in hepcidin production could result in higher ferritin levels seen in subjects with MetS. However, althought reduced adipose tissue oxygenation was found in obese subjects, hepcidin expression levels were increased, not suppressed, in the adipose tissue of obese patients [27]. Consequently, it is unlikely that purely adipose tissue hypoxia could cause hepcidin supression and elevated ferritin levels.

Haptoglobin is an acute phase reactant which plasma levels are increased during inflammation [28]. Although the liver is the major source of haptoglobin, research has demonstrated that it is also secreted into plasma by adipose tissue [29]. Serum haptoglogin level was previously shown to be positively associated with body fat [30]. Our study shows higher serum haptoglobin levels in subjects with MetS and subjects with elevated glucose or blood pressure component even after adjusting for hs-CRP.

Serum transferrin receptor levels did not differ between subject with or without MetS, but sTFR level was higher in subjects with abdominal obesity component of MetS. sTFR levels are increased in iron defiency with inadequate iron supply for erythropoiesis [31] but also, for example secondary to use of erythropoiesis stimulating agents such as erythropoietin [32]. Higher sTFR levels in subjects with abdominal obesity component of MetS suggest that despite of higher ferritin levels, these subjects are not iron overloaded. Previous studies have shown higher sTFR leves in obese subjects as well as no iron accumulation in liver biopsies of obese patients [27,33].

The strength of our study is the study population with five age groups and no exclusion criteria. Smoking habits between subjects with and without MetS did not differ significantly. In addition, all results were adjusted for smoking to exclude its influence particularly on hemoglobin and erythropoietin levels. All results were also hs-CRP adjusted to estimate the impact of inflammation. A limitation is that hematological parameters were measured only at the second health check-up and cross-sectional study design does not allow identification of proper causal relationships. Information about women’s menopause status was not available. However, the separate analysis was done in women for age adjusment (Table 1, all data not shown). The age adjusment did not affect the results in women. Unfortunately, we were unable to evaluate a possibly impact of obstructive or central sleep apnea on subjects hemoglobin or erythropoietin levels. Also, information about nutritional content of subjects’ diets or consumption of dietary supplements like iron or antioxidants was not available.

In conclusion, Subjects with MetS have elevated hemoglobin, ferritin, erythropoietin and haptoglobin concentrations. Higher hemoglobin levels are related to all components of MetS. Higher ferritin levels associate with TG, abdominal obesity, elevated glucose or low high-density cholesterol. Haptoglobin levels associate with blood pressure or elevated glucose. However, erythropoietin levels are related only with abdominal obesity. Higher serum erythropoietin concentrations may suggest underlying adipose tissue hypoxemia in MetS.

## Competing interests

The authors declare that they have no competing interests.

## Authors’ contributions

PH wrote the manuscript. JS contributed to discussion and reviewed and edited the manuscript. HK performed the statistical analyses and reviewed and edited the manuscript. PM contributed the discussion and reviewed and edited the manuscript. MV researched data, contributed to discussion and reviewed and edited manuscript. All authors read and approved the final manuscript.
